# Function of alanine racemase in the physiological activity and cariogenicity of *Streptococcus mutans*

**DOI:** 10.1038/s41598-018-24295-1

**Published:** 2018-04-13

**Authors:** Shiyu Liu, Yuan Wei, Xuedong Zhou, Keke Zhang, Xian Peng, Biao Ren, Vivian Chen, Lei Cheng, Mingyun Li

**Affiliations:** 1 State Key Laboratory of Oral Diseases, West China Hospital of Stomatology, Sichuan University, NO. 14, 3rd Section of South RenMin Rd, Chengdu, Sichuan 610041 China; 2 Department of Operative Dentistry and Endodontics, West China Hospital of Stomatology, Sichuan University, Chengdu, Sichuan China; 3 Department of Endodontology, Nanjing Stomatological Hospital, Medical School of Nanjing University, NO. 30 Zhongyang Road, Nanjing, 210008 China; 4 Columbia University, New York, USA

## Abstract

The enzyme alanine racemase (Alr) has been a new target for the development of antibacterial drugs based on the involvement of D-Ala in bacterial cell wall biosynthesis. Our previous study noted that Alr is essential for the growth and interspecies competitiveness of *S*. *mutans*, the major causative organism of dental caries. However, physiological activity and cariogenicity of *S*. *mutans* affected by Alr remains unknown. The current study examined the biofilm biomass, biofilm structure, extracellular polysaccharide (EPS) synthesis, glucosyltransferase (*gtf*) gene expression, acid production and acid tolerance in the *alr*-mutant strain. We found that biofilm formation, biofilm structure, and EPS synthesis was in a D-Ala dose-dependent manner. Biofilm structure was loose in *alr*-mutant group and the ratio of EPS/bacteria was also elevated. Additionally, the expression levels of multiple *gtfs* were up-regulated, and acid tolerance was decreased. We also established *in vivo* models of dental caries and found that the incidence and severity of the caries were decreased in the *alr*-mutant group in comparison to the parental *S*. *mutans* group. Our *in vivo* and *in vitro* experiments demonstrate that Alr is essential for the cariogenicity of *S*. *mutans* and that Alr might be a potential target for the prevention and treatment of caries.

## Introduction

Alanine racemase (Alr) is a bacterial enzyme that catalyses the conversion of L-alanine to D-alanine (D-Ala)^1^. This function is critical for the growth of bacteria due to their need for D-alanine, an essential component in the biosynthesis of cell wall peptidoglycan in both gram-positive and gram-negative bacteria^2^. Two kinds of Alr have been identified in bacteria: the *alr*-encoded racemase, which is constitutive and used for D-Ala biosynthesis, and the *dadX*-encoded racemase, which is inducible and used for the catabolism of D-Ala^1,3,4^. Lack of expression of the *alr* gene is lethal when there is no addition of exogenous D-Ala^5^. Many studies have focused on alanine racemase, which aim to develop antibacterial drugs for multiple bacterial species, such as *Pseudomonas aeruginosa*^6^, *Acinetobacter baumannii*^7^, *Staphylococcus aureus*^8^, *Bacillus anthracis*^9^, *Lactobacillus plantarum*^10^, and *Escherichia coli*^11^.

Dental caries is one of the most common chronic diseases worldwide and is considered a major health concern for professionals^12^. It is the main cause of tooth pain, tooth loss and can affect overall health by making food intake more difficult^13^. Therefore, focusing on the factors affecting caries progression and finding efficient strategies for the management of caries is of great significance. *Streptococcus mutans* (*S*. *mutans*) is the dominant bacterium in dental plaque and has long been implicated as the major causative organism of dental caries^14^. *S*. *mutans* is able to ferment carbohydrates and produce acids that reduce the local pH^15^. In addition, *S*. *mutans* embedded in the dental plaque is resistant to host defences because the extracellular matrix inside the biofilms provides protection from harmful factors^16^. Exploring factors that influence the cariogenic virulence of *S*. *mutans* might aid in the discovery of a more effective target for an anti-caries drug. Considering the essential role of D-alanine for the bacterial cell wall, the *alr* gene is a potential antibacterial target for *S*. *mutans*.

In our previous study, an *alr*-mutant strain was constructed, and the role of *alr* in cell growth and cell wall integrity was explored. Our data showed that a minimal concentration of D-Ala (150 µg/ml) was required for the optimal growth of the *alr*-mutant strain. The depletion of D-Ala in the growth medium led to cell wall perforation and cell lysis in the *alr*-mutant strain^17^. Another previous study also demonstrated the important role of D-Ala metabolism for the growth of *S*. *mutans*^18^. Given the importance of *alr* to the growth of *S*. *mutans*, the aim of this study was to explore the role of Alr on the physiological activity, including biofilm formation, extracellular polysaccharide (EPS) synthesis, glucosyltransferases (*gtfs*) gene expression, acids production and tolerance, and cariogenicity of *S*. *mutans*.

## Results

### A D-Ala dose-dependent manner in *alr*-mutant biofilm formation

**Figure 1:**
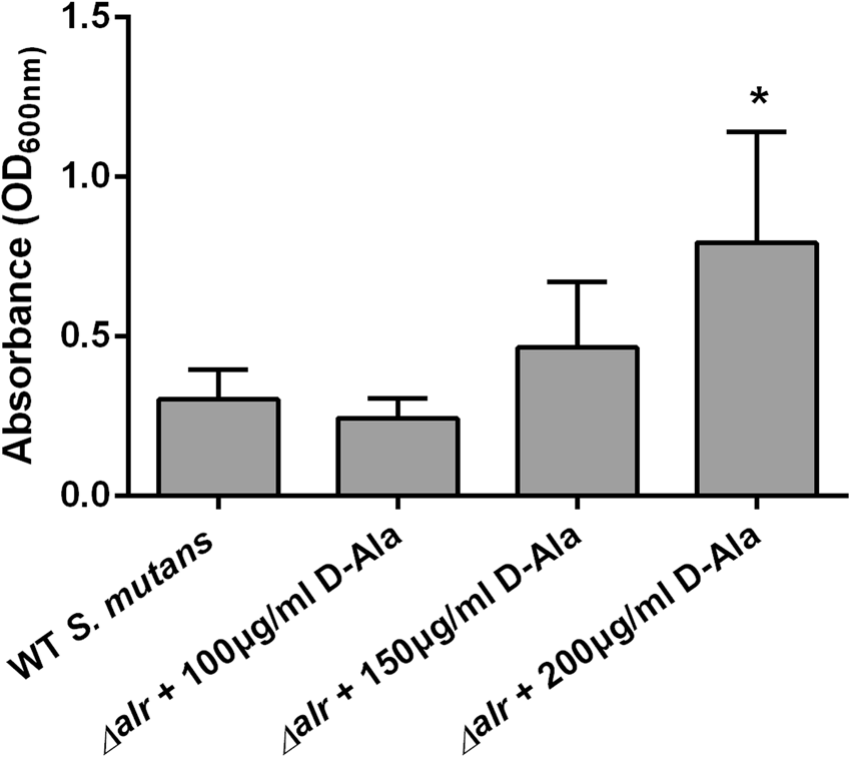
Biofilm biomass assay by crystal violet staining. The absorbance of the crystal violet-stained *S*. *mutans* biofilm at 600 nm is shown with the mean plus standard deviation (SD). The asterisks indicate the significant differences compared to the parental *S*. *mutans* strain group. The error bars represent the SD. *P < 0.05.

We have studied the growth of WT *S*. *mutans* affected by exogenous D-Ala, and the result showed that exogenous D-Ala did not obviously affect the growth of *S*. *mutans* (see Supplementary Fig. S1). And we also have determined the biofilm biomass by crystal violet staining of the WT *S*. *mutans* affected by different concentrations of exogenous D-Ala, and the result showed no significantly difference either (see Supplementary Fig. S2). Therefore, the addition of D-Ala was a complementation to the *alr*-mutant. As shown in Fig. 1, Alr is important for *alr*-mutant strain biofilm formation and biofilm biomass increased in a D-Ala dose-dependent manner. Compared to the parental *S*. *mutans* group, biofilm biomass was higher in 200 μg/ml of D-Ala with statistical significance. However, there was no obvious difference among the other two *alr*-mutant groups and WT *S*. *mutans* group.

### Loose biofilm structure and short planktonic cells chain of *alr*-mutant strain

**Figure 2:**
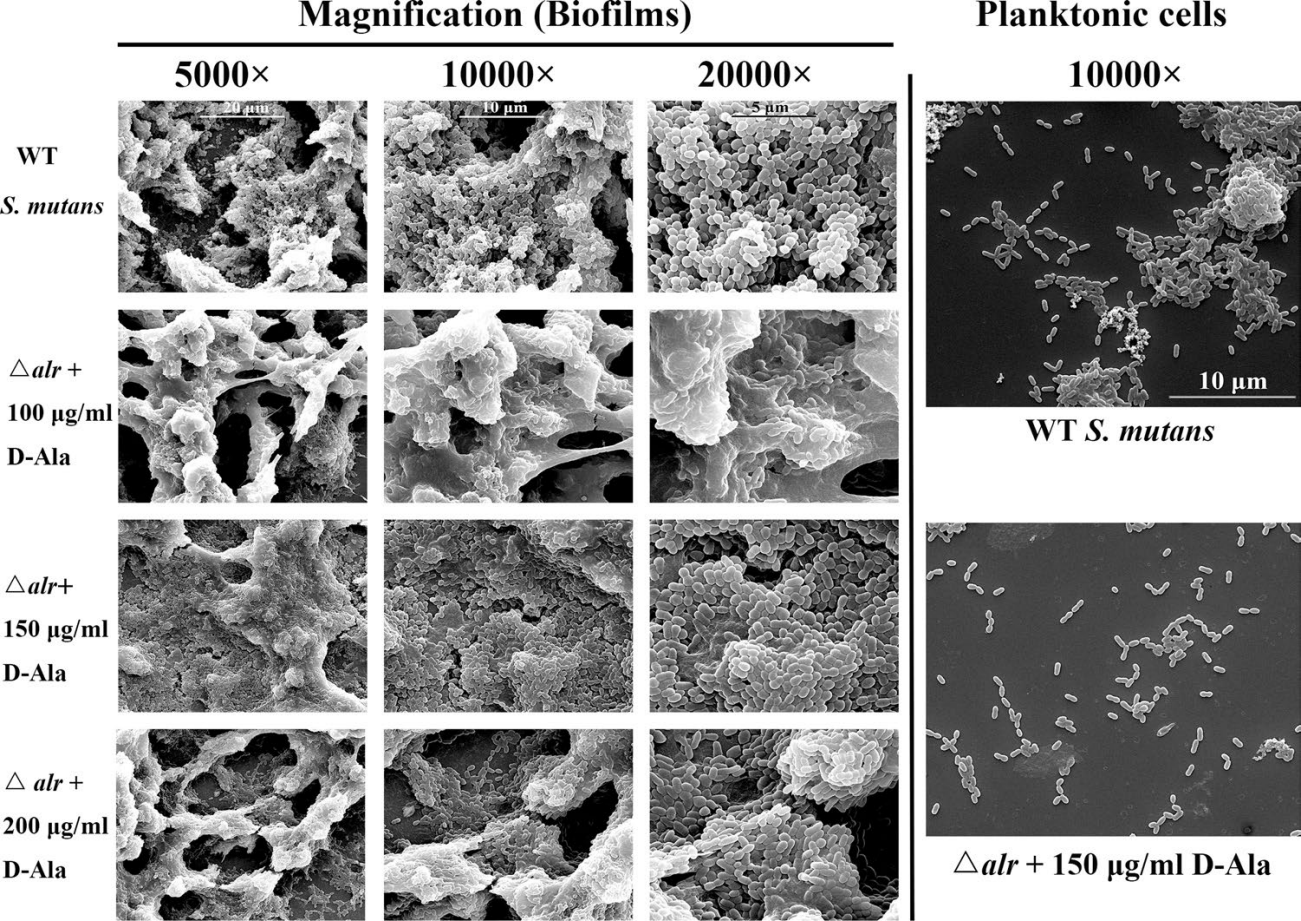
Scanning electron microscopy images of biofilms and planktonic cell morphology. Biofilm images were obtained at 5000×, 10000× and 20000×. Planktonic cell images were obtained at 10000×.

Compared to parental *S*. *mutans* group (Fig. 2), the biofilm structure was loose at a concentration of 100 µg/ml of D-Ala, and there were fewer cells but more extracellular matrix within the biofilms. Biofilms became denser as D-Ala concentration increased (150 μg/ml, 200 μg/ml). The morphology of the planktonic cells supplemented with 150 µg/ml of D-Ala was also recorded by a scanning electron microscopy; the *alr*-mutant cells were more randomly distributed and showed a shorter chain length than the parental *S*. *mutans* strain.

### Increased EPS synthesis and decreased bacterial cell numbers in the *alr*-mutant group according to CLSM

**Figure 3:**
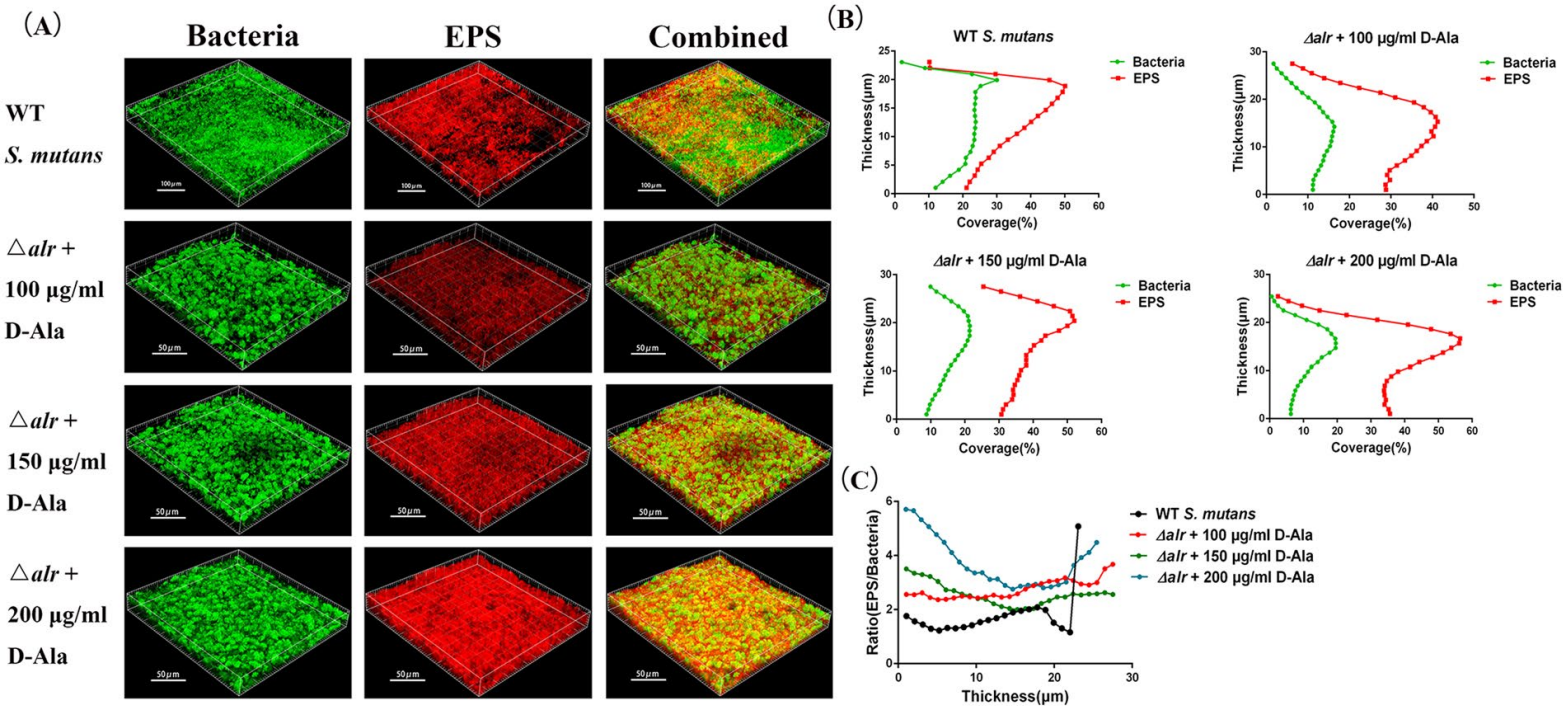
Bacterial cell multiplication and EPS synthesis by confocal laser scanning microscopy (CLSM). (**A**) The three-dimensional reconstruction of biofilms. Reconstruction of the biofilms was performed with IMARIS 7.0. Bacterial cells were labelled with the SYTO 9 green fluorescent dye (left column), and EPS was labelled with the Alexa Fluor 647 red fluorescent dye (middle column). (**B**) The EPS and bacteria distributions on the reconstructed biofilm. (**C**) The EPS/bacteria ratio. The asterisks indicate significant differences compared to the parental *S*. *mutans* strain group. The error bars represent the standard deviation (SD). *P < 0.05.

As EPS are major components of *S*. *mutans* biofilms, we captured images by confocal laser scanning microscopy and performed three-dimensional reconstructions to explore how EPS were affected by Alr. As shown in Fig. 3A, EPS synthesis was markedly enhanced in a D-Ala-dose-dependent manner. At a D-Ala concentration of 100 µg/ml, the EPS around the bacterial cells were rare and became more abundant in the 200 µg/ml group. However, we found that the bacterial cell numbers in three *alr*-mutant groups were less than cell numbers in the parental *S*. *mutans* group (Fig. 3B). The coverage of wild type *S*. *mutans* cells was over 20% in most layers while the coverage of *alr*-mutant strain was less than 20%. The distance between the red line and green line represents the relative ability of bacteria producing EPS. It shows that the *alr*-mutant strain produced more EPS compared to the parental *S*. *mutans* strain. Moreover, the ratio of EPS/bacterial cells formed by the *alr*-mutant strain was also higher than that of the parental *S*. *mutans* strain (Fig. 3C).

### Up-regulated expression of EPS synthesis-associated genes in the *alr*-mutant group according to qRT-PCR analysis

**Figure 4:**
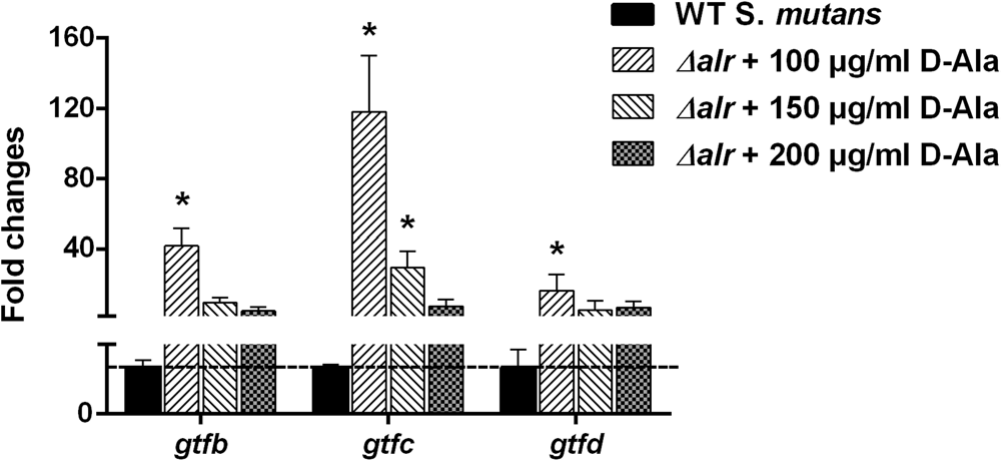
qRT-PCR assays for the *gtfs* gene expression of WT *S*. *mutans* and *alr*-mutant. Expression of *gtfs* genes. The asterisks indicate significant differences compared to the parental *S*. *mutans* strain group. The error bars represent the standard deviation (SD). *P < 0.05.

Compared to the parental *S*. *mutans* strain, the expression of *gtfB*, *gtfC*, and *gtfD* in the *alr*-mutant cells was up-regulated in the three different D-Ala concentration groups. However, there was no statistical significance in the 200-µg/ml group, and only *gtfC* gene expression significantly increased (by 29.3-fold) in the 150 µg/ml group. Nevertheless, the mRNA levels of *gtfB*, *gtfC*, and *gtfD* in the *alr*-mutant cells of the 100-µg/ml group were significantly up-regulated by 41.7-, 117.9-, and 16.2-fold, respectively (Fig. 4).

### Decreased acid tolerance in *alr*-mutant strain

**Figure 5:**
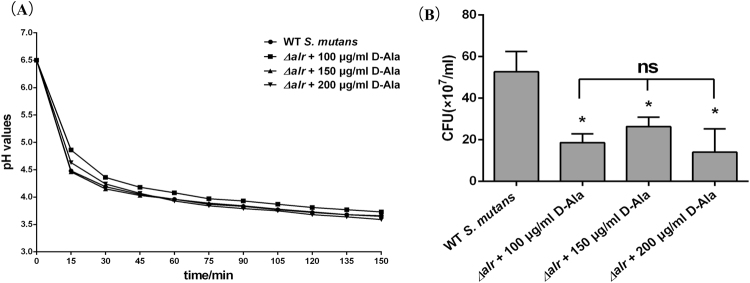
Acid production (**A**) and acid tolerance (**B**) of the *alr-mutant* strain compared to the parental *S*. *mutans* strain. The asterisks indicate significant differences compared to the parental *S*. *mutans* strain group. The error bars represent the standard deviation (SD). *P < 0.05, ns: no significance.

There was no obvious difference in acid production among *alr*-mutant groups and wild type *S*. *mutans* group (Fig. 5A). However, acid tolerance was significantly decreased in the absence of the *alr* gene (Fig. 5B). Fewer bacterial colonies were formed by the *alr*-mutant groups than by the control group. No statistically significant differences were observed for acid tolerance between the three *alr*-mutant groups that had been supplemented with different D-Ala concentrations.

### Decreased cariogenicity of *alr*-mutant strain in rats

The caries lesions of each rat were scored and recorded according to the Keyes scoring system, which divides lesions into four grades: enamel only (E), slightly dentinal (Ds), moderate dentinal (Dm), and extensive dentinal (Dx). Ds lesions represent the involvement of 1/4 of the dentin between the enamel and the pulp chamber. Dm lesions represent the involvement of 1/4 ~ 3/4 of the dentin region. Dx lesions represent caries progression beyond 3/4 of the dentin region.

**Figure 6:**
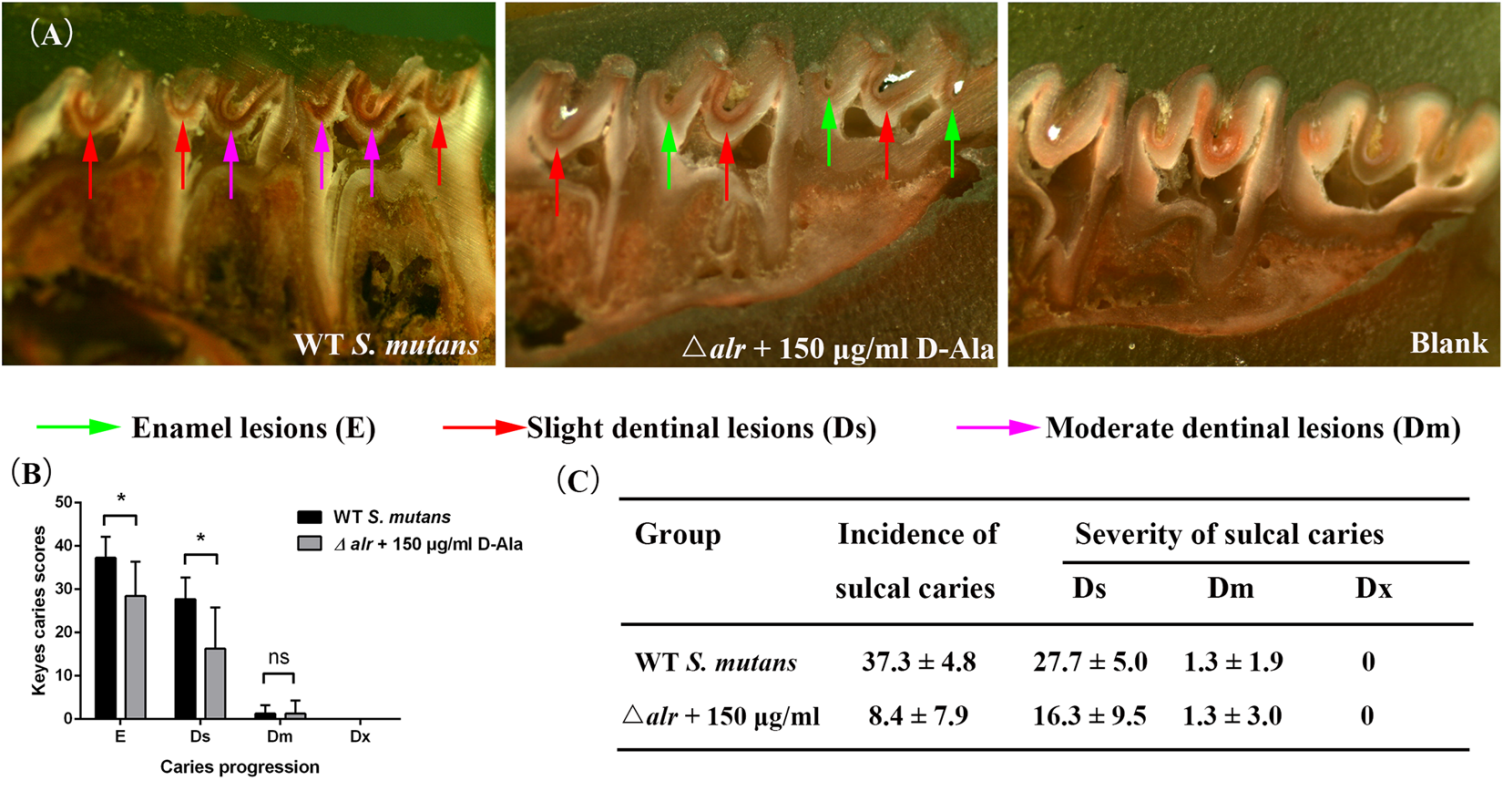
Caries lesions in rats challenged with the wild type *S*. *mutans* or *alr*-mutant strain. (**A**) Stereo microscopy images of caries lesions. (**B**) Statistical analysis of the Keyes scores. The asterisks indicate significant differences compared to the parental *S*. *mutans* strain group. The error bars represent the standard deviation (SD). *P < 0.05. (**C**) The scores for the enamel lesions (E) represent the incidence of caries. The scores for the slightly dentinal lesions (Ds), moderate dentinal lesions (Dm), and extensive dentinal lesions (Dx) represent the severity of the caries.

Photographs of the caries lesions on the teeth of the rats were obtained using a stereo microscope (Fig. 6A). No caries lesions were observed in the blank group. In the wild type *S*. *mutans* group, the range of caries lesions was larger and deeper in depth as most of the sulcal caries had progressed to the slight dentine (Ds); a few moderate dentinal lesions (Dm) were also observed. The 150 µg/ml D-Ala *alr*-mutant group, however, had fewer Ds lesions.

The statistical analysis is shown in Fig. 6B. More E and Ds lesions were observed in the *S*. *mutans* group (P < 0.05) than the *alr*-mutant group. In addition, no Dx lesions occurred in either group. Figure 6C shows that there was an obvious decrease in the incidence and severity of sulcal caries in the *alr*-mutant strain group.

## Discussion

To explore whether Alr can represent an effective drug target to modulate the cariogenicity of oral biofilm and benefit the management of dental caries, we firstly determined that Alr is essential for the growth and interspecies competitiveness of *S*. *mutans*^17^. A minimal concentration of D-Ala (150 µg/ml) was required for the optimal growth of the *alr*-mutant strain. D-Ala starvation in the growth medium led to cell wall perforation and cell lysis in the *alr*-mutant strain. Although Alr has been well demonstrated to be closely associated with the survival and cell wall in *S*. *mutans*, its cariogenicity and physiological importance in biofilm is yet to be explored. In the current study, we examined the effect of Alr on *S*. *mutans* biofilm formation, biofilm structure, EPS synthesis, the expression of genes involved in sugar production, acid production, and acid tolerance, all of which are responsible for caries development. We also observed that Alr is closely associated with caries development. Similar D-Ala dose-dependent manner was observed in *alr*-mutant biofilm formation, biomass and EPS synthesis, which suggested *alr* is important for the mutant biofilm structure.

The loose biofilm structure of the *alr*-mutant was observed at a concentration of 100 µg/ml of D-Ala. The biofilms became denser as the D-Ala concentration increased. The observed biofilm change may also be attributed to the mutant death and cell lysis we have reported previously. A pronounced short chain length of *alr*-mutant compared to the wild type under the scanning electron microscopy also supported the possibility.

Interestingly, we also observed that the *alr*-mutant produced more EPS compared to the parental *S*. *mutans* strain with fewer cells in most layers of the biofilm. The EPS/bacteria ratio of *alr*-mutant was elevated, and the elevated ratio was consistent with the qPCR results of the expression levels of the *gtfB*, *gtfC*, and *gtfD* genes, which might be explained by the regulatory systems inside *S*. *mutans* such as two-component signal transduction systems (TCSTS). Some TCSTS e.g. VicRK and CovR are responsible for sucrose-dependent adherence and biofilm formation^19,20^. The expression of *gtfB/C/D* is positively regulated by VicR and/or repressed by CovR^20^. The cell lysis of the *alr*-mutant was sensed by TCSTS, which subsequently turned on the up-regulation pathway to EPS synthesis for survival as high EPS levels lead to a thick biofilm, causing resistance to immune factors or harmful metabolites^16^. There was no adequate D-Ala in the 100 μg/ml group, and *alr*-mutant exhibited strongest EPS-producing ability, indicating stronger survivability in the worst environment. Therefore *gtfs* expression was highest at 100 μg/ml. However, EPS synthesis was highest in the mutant treated with 200 μg/ml D-Ala. We speculate that the high concentration of D-Ala served as an additional carbon source that the bacteria utilized to produce polysaccharide. As previously mentioned, *S*. *mutans* is able to metabolize carbohydrates that leads to acid accumulation and a subsequent pH declination in the dental biofilm^21^. EPS provide a limiting barrier for acid diffusion by generating acidic microenvironments within the dental plaque. This dual-effect leads to a continuous pH decline to the critical pH, below which tooth hard-tissue demineralization begins and dental caries occurs^22,23^. Although the *alr*-mutant produced high EPS levels and limited acid diffusion, the distinctly weak acid tolerance (Fig. 5B) also limited the acid accumulation and continuous pH declination. *Alr* significantly affected acid tolerance, which we speculate is caused by cell wall instability in the *alr*-mutant strain, even though there was adequate D-Ala supplied in the culture medium. This hypothesis, however, needs to be verified in a further study. The exact mechanisms are still unknown, but deletion of *alr* affects the formation as well as structure of the biofilm, indicating that *alr* plays an important role in the physiological activity of *S*. *mutans*.

To better understand the exact role of Alr in the cariogenicity of *S*. *mutans*, we developed the caries rat model and found that the caries incidence and severity in the *alr*-mutant group were lower than in the parental *S*. *mutans*. Although EPS synthesis and *gtfs* expression were increased in the *alr*-mutant in introductory studies, caries lesions still decreased in the *alr*-mutant. This might be due to the poor acid tolerance of the *alr*-mutant strain. There were no sufficient living cells within biofilms and thus no sufficient acid accumulation. These data indicate the compromised cariogenicity of the *alr*-mutant, further supporting our hypothesis that Alr could be a promising target to control the prevalence of cariogenic of *S*. *mutans*.

In conclusion, the current study demonstrated that Alr is important for the physiological activity and cariogenicity of the S. mutans. Alr can represent a promising target for the management of dental caries.

## Materials and Methods

### Chemicals, bacterial strains and growth conditions

*S*. *mutans* UA 159 was obtained from the American Type Culture Collection (Manassas, VA, USA) and was routinely cultured in brain heart infusion broth (BHI; Difco, Sparks, MD, USA) at 37 °C aerobically (95% air/5% CO_2_). The *alr*-mutant strain^17^ was cultured in BHI plus 100, 150, or 200 µg/ml of D-Ala (Sigma). The medium was supplemented with 1% sucrose (called BHIS) when needed. 1 × 10^7^ CFU/ml of *S*. *mutans* or the *alr*-mutant strain was used for both *in vitro* and *in vivo* studies.

### Crystal violet staining for biofilm biomass analysis

After culturing in 96-well microtiter plates for 24 h, the biofilms were gently washed three times with phosphate buffer saline (PBS), fixed with 95% methanol for 30 min, washed three times with PBS, stained with 0.5% crystal violet for 30 min and washed three times with PBS. The crystal violet was extracted with 200 µl of 100% ethanol. The extract was evaluated at 600 nm using a spectrophotometer^24^.

### Scanning electron microscopy (SEM) analysis of biofilm and planktonic cell morphology

After culturing in 24-well microtiter plates for 24 h, the biofilms (grown on glass slides) were gently washed three times with PBS, fixed with glutaraldehyde (2.5%) overnight at 4 °C, washed three times with PBS, dehydrated using a series of ethanol rinses (30, 50, 70, 80, 85, 90, 95 and 100%), immersed for 10 min in 100% ethanol and dried in a desiccator^24,25^. For planktonic cells, cells were pelleted by centrifugation at 4000 rpm for 5 min, washed twice with PBS and re-suspended. An aliquot (5.0 µl) of bacterial suspension was deposited on a sterile glass slide and air-dried at 37 °C. Samples were then fixed, dehydrated, immersed and dried^26^. After coating with gold-palladium, samples were analysed in a scanning electron microscope (Inspect F; FEI, Eindhoven, The Netherlands) at 5000×, 10000×, 20000× magnification.

### Confocal laser scanning microscopy (CLSM) for the assessment of extracellular polysaccharide (EPS) synthesis within biofilms

Biofilms cultured on glass slides were grown in BHIS medium and supplemented with 1 µM Alexa 647 red fluorescent dye (Molecular Probes Inc., OR, USA) and protected from light. Alexa 647 are labelled dextran conjugates and were incorporated into EPS. After incubation for 24 h, the biofilms were washed with PBS three times and incubated with 1 µM SYTO 9 green fluorescent dye (Molecular Probes Inc., OR, USA) at room temperature for 20 min. SYTO 9 bound to bacterial nucleic acid and represented bacteria in biofilms. Then the biofilms were washed with PBS three times and dried. ProLong Gold Antifade Reagent was dropped on the biofilms, and images were obtained using a confocal laser scanning microscope (TCS SP2; Leica Microsystems, Wetzlar, Hessen, Germany). The Alexa Fluor 488 and 647 fluorescent channels were selected to detect green and red fluorescence respectively. The whole procedure was performed away from light^27,28^.

### Quantitative real-time PCR (qRT-PCR) analysis of *S*. *mutans* EPS synthesis-associated genes

The qRT-PCR protocol has been described in detail in our previous studies^29–31^. Briefly, the biofilms were harvested after incubation in BHIS medium for 24 h, washed three times with PBS, and stabilized using the RNAprotect Bacteria Reagent (1 ml, Qiagen, MD, USA). Then, the biofilms were suspended in 180 µl of lysis buffer (containing 20 mM Tris-HCl, pH 8.0, 1.2% Triton-100, 1 mM EDTA-Na_2_, and 30 mg/ml of lysozome) with 10 µl of mutanolysin (10 KU/ml, Sigma-Aldrich, MO, USA) and 15 µl of proteinase K (Qiagen), incubated with agitation at 37 °C for 120 min, and sonicated for 5 cycles (10 s/cycle, 52% amplitude, Sonic Dismembrator, Model 500, Fisher Scientific). The biofilm RNA was extracted and purified using the RNeasy Mini kit (Qiagen). The RNA concentration was measured using a NanoDrop 2000 (Thermo Fisher Scientific Inc., USA). A high-capacity cDNA reverse transcription kit with random primers (Applied Biosystems, Life Technologies Corp., CA, USA) was used to synthesize cDNA from 10 µg RNA. PCR reactions comprised two micrograms of cDNA, *S*. *mutans* primers (0.375 µM) and the Fast SYBR green master mix (Applied Biosystems) and were run using an ABI Prism 7000 sequence detection system for quantitative PCR. The primers were 16 S, *gtfB*, *gtfC*, and *gtfD*^31^. The values of 2^−∆∆Ct^ were analysed to calculate the gene expression fold changes^21^.

### Glycolytic pH drop assay for acid production

The role of Alr in *S*. *mutans* glycolysis was measured using the method described by Xu *et al*.^32^. The bacteria were harvested at the mid-logarithmic growth phase, washed with PBS and resuspended (OD_600 nm_ = 0.5) in 0.5 mM potassium phosphate buffer consisting of 1.25 mM MgCl_2_ and 37.5 mM KCl (pH = 6.5). Glucose was added to the bacterial suspension, and the final concentration was adjusted to 1% (wt/vol). The glycolytic activity of *S*. *mutans* and the *alr*-mutant strains resulted in a decrease in pH, and the pH levels were monitored at 15-min intervals over a period of 120 min.

### Acid tolerance assay

The role of Alr in *S*. *mutans* acid tolerance was also measured using the method described by Xu *et al*.^32^. The bacteria were harvested at the mid-logarithmic growth phase, collected by centrifugation and resuspended (OD_600 nm_ = 0.2) in TYEG medium (containing 10% tryptone, 5% yeast extract, 3% K_2_HPO_4_, and 1% glucose) buffered with 40 mM phosphate-citrate buffer solution (pH = 5.0) and incubated at 37 °C aerobically for 2 h. After incubation at pH 5.0, samples were removed for viable counts.

### *In vivo* models of dental caries

The *in vivo* study was approved by the ethics committee of West China School of Stomatology, Sichuan University (WCCSIRB-D-2014-072), and all experiments were performed according to the National Institutes of Health Guide for the Care and Use of Laboratory Animals.

Sixteen specific pathogen-free (SPF) male Wistar rats, aged 21 days, were randomly divided into three groups: *S*. *mutans* (n = 7), *alr*-mutant (150 µg/ml, n = 7), and blank (n = 2). Upon arrival, animals were determined to be free of any indigenous oral microorganism by feeding the rats antibiotics, namely, ampicillin, chloramphenicol and carbenicillin (1.0 g/kg), for 3 consecutive days^33^. After a washout period of 3 days, in which the residual antibiotics in the oral cavity were removed by feeding rats antibiotic-free distilled water, rats were challenged with 1.0 × 10^7^ CFU/ml *S*. *mutans* or *alr*-mutant strain suspensions for 3 consecutive days (twice per day at 30-min intervals with no food or water for 1 h after incubation). All rats were fed the National Institutes of Health cariogenic diet 2000 and 5% sucrose water^34^. For the *alr*-mutant group, D-Ala was added to the water (150 µg/ml). The experiment lasted for 27 days, after which the rats were sacrificed. The jaws of the rats were aseptically dissected, and the caries status was scored using the Keyes method^35^.

### Statistical Analysis

Each experiment was independently repeated at least three times. One-way analysis of variance (ANOVA) was used to analyse the crystal violet staining, acid tolerance, and qRT-PCR. Independent t-tests were used for the *in vivo* study. The data were statistically analysed using the SPSS 21.0 software. P < 0.05 was considered statistically significant.

### Data Availability

The datasets generated and analysed during the current study are available from the corresponding authors on reasonable requests.

## Electronic supplementary material

Supplementary information
