# Supplementary material for: Function of alanine racemase in the physiological activity and cariogenicity of Streptococcus mutans
Source: Sci Rep. 2018 Apr 13;8:5984. doi: 10.1038/s41598-018-24295-1 (PMC5899142; doi:10.1038/s41598-018-24295-1)
Supplement: Supplementary file 1 — Supplementary information [file 41598_2018_24295_MOESM1_ESM.pdf]

Function of alanine racemase in the physiological activity and cariogenicity of *Streptococcus mutans*

Shiyu Liu#, Yuan Wei#, Xuedong Zhou, Keke Zhang, Xian Peng, Biao Ren, Vivian Chen, Lei Cheng*, Mingyun Li1*

Corresponding author:

Mingyun Li. Email: limingyun@scu.edu.cn

Lei Cheng. Email: chenglei@scu.edu.cn

This PDF file includes:

Fig. S1. The growth curve of WT *S. mutans* with different concentrations of D-alanine (100, 150 and 200 μg/ml) for 24 h.

Fig. S2. The biofilm formation of WT *S. mutans* with different concentrations of D-alanine (100, 150 and 200 μg/ml) for 24 h.

Methods

Planktonic growth of WT *S. mutans*

For planktonic growth curve assays of WT *S. mutans*, overnight bacterial cultures were added to BHI containing different concentration of D-Ala (100, 150 and 200 μg/ml). Two hundred microlitre of 1×107 CFU/ml of *S. mutans* were put into selected wells of a sterile 96-well microtiter plate and incubated at 37 ℃ in air (keep out of uncontaminated) for 24 h. The turbidity was measured by optical density (OD) at 600 nm using a spectrophotometer every 1 hour [1].

Crystal violet staining for WT *S. mutans* biofilm biomass analysis

Two hundred microlitre of 1×107 CFU/ml of WT *S. mutans* were cultured in BHI medium plus 1% sucrose containing different concentration of D-Ala (100, 150 and 200

μg/ml). After culturing in 96-well microtiter plates for 24 h, the biofilms were gently washed three times with phosphate buffer saline (PBS), fixed with 95% methanol for 30 min, washed three times with PBS, stained with 0.5% crystal violet for 30 min and washed three times with PBS. The crystal violet was extracted with 200 μl of 100% ethanol. The extract was evaluated at 600 nm using a spectrophotometer [2].

Results

Exogenous D-Ala did not obviously affect the growth and biofilm formation of WT *S. mutans* at the current D-Ala concentration (Fig. S1 & S2). There is no statistical difference in biofilm biomass between four groups.

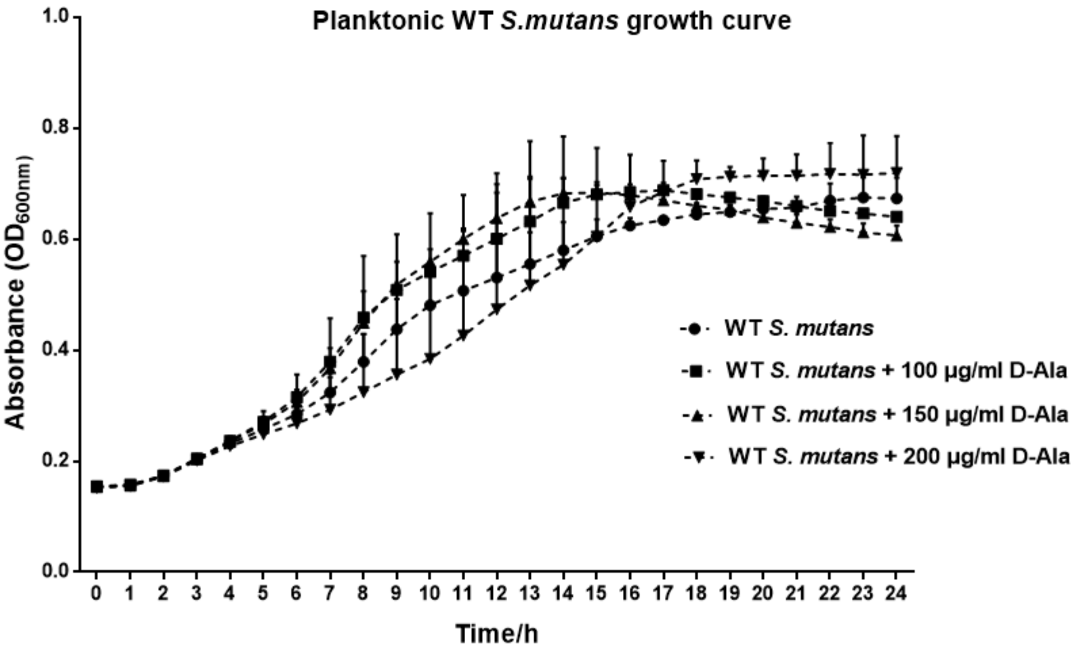

Fig. S1. The growth curve of WT *S. mutans* with different concentrations of D-alanine (100, 150 and 200 μg/ml) for 24 h. The absorbance at 600 nm is shown with the mean plus standard deviation (SD).

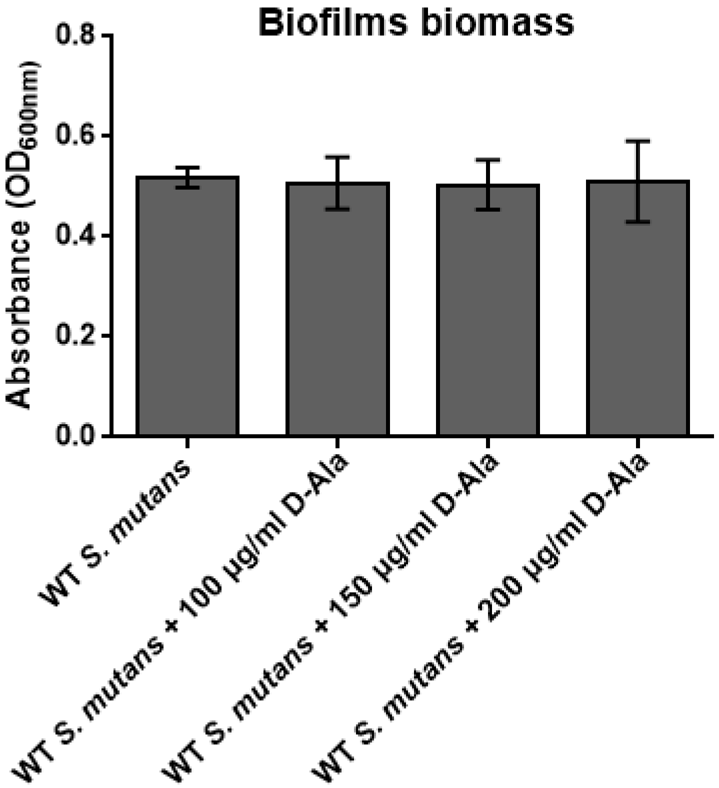

Fig. S2. The biofilm formation of WT *S. mutans* with different concentrations of D-alanine (100, 150 and 200 µg/ml) for 24 h. The absorbance of the crystal violet-stained *S. mutans* biofilm at 600 nm is shown with the mean plus standard deviation (SD).

References:

1. Li, M. et al. Effect of nicotine on dual-species biofilms of *Streptococcus mutans* and *Streptococcus sanguinis*. *FEMS Microbiol Lett* **350**, 125–132; 10.1111/1574-6968.12317 (2014).

2. Huang, R., Li, M., Gregory, R. L. Effect of nicotine on growth and metabolism of *Streptococcus mutans*. *Eur J Oral Sci* **120**, 319-325; 10.1111/j.1600-0722.2012.00971.x (2012).
